# Post-Translational Modifications (PTMs) of mutp53 and Epigenetic Changes Induced by mutp53

**DOI:** 10.3390/biology13070508

**Published:** 2024-07-08

**Authors:** Rossella Benedetti, Michele Di Crosta, Gabriella D’Orazi, Mara Cirone

**Affiliations:** 1 Department of Experimental Medicine, Sapienza University of Rome, Viale Regina Elena 324, 00161 Rome, Italy; rossella.benedetti@uniroma1.it (R.B.); michele.dicrosta@uniroma1.it (M.D.C.); 2 Department of Neurosciences, Imaging and Clinical Sciences, University “G. D’Annunzio”, 66013 Chieti, Italy

**Keywords:** mutant p53, phosphorylation, ubiquitination, methylation, epigenetic changes

## Abstract

**Simple Summary:**

Post-translational modifications (PTMs) strongly influence the stability and function of proteins. These modifications have been reported to affect wild-type (wt) p53 as well as the mutant forms of this protein (mutp53), often detected in cancer cells. Thus, PTMs may be key regulators of the oncosuppressor activity of wtp53 and of the pro-oncogenic functions that some mutant forms of p53 may acquire, in terms of tumor survival, progression and resistance to anti-cancer therapies. As treatments that specifically target mutp53 do not exist, manipulating PTMs may represent a promising approach to achieve this goal and even to reactivate the wt functions of mutant proteins, in some cases, as reported in this review.

**Abstract:**

Wild-type (wt) p53 and mutant forms (mutp53) play a key but opposite role in carcinogenesis. wtP53 acts as an oncosuppressor, preventing oncogenic transformation, while mutp53, which loses this property, may instead favor this process. This suggests that a better understanding of the mechanisms activating wtp53 while inhibiting mutp53 may help to design more effective anti-cancer treatments. In this review, we examine possible PTMs with which both wt- and mutp53 can be decorated and discuss how their manipulation could represent a possible strategy to control the stability and function of these proteins, focusing in particular on mutp53. The impact of ubiquitination, phosphorylation, acetylation, and methylation of p53, in the context of several solid and hematologic cancers, will be discussed. Finally, we will describe some of the recent studies reporting that wt- and mutp53 may influence the expression and activity of enzymes responsible for epigenetic changes such as acetylation, methylation, and microRNA regulation and the possible consequences of such changes.

## 1. Introduction

The proper functioning of wild-type p53 (wtp53) is irreconcilable with cancer onset, and indeed, it is the most frequently mutated or inactivated tumor suppressor gene in human cancers [1]. In addition to reducing the genome’s guardian properties, growing evidence suggests that mutations in the gene encoding p53 may turn this tumor suppressor into an oncogene [2]. Several p53 mutations have been identified in tumor cells in the context of different tumors, including but not limited to P98S, P151H, A161T, R175C, R175D, R175H, S227K, S227R, G245C, R248L, R248W, R273H, R273L, and R280K. Some of them are considered hot-spot and missense mutations (e.g., R175H and R273H) because they occur in residues critical for p53 function. In some cases, mutant p53 (mutp53) may acquire gain-of-function (GOF) properties, leading to disastrous consequences for cells harboring these mutations [3,4]. mutp53 can engage in cross-talk with several pathways and play a key role in driving carcinogenesis, such as signal transducer and activator of transcription 3 (STAT3), mechanistic target of rapamycin (mTOR), nuclear factor kappa-light-chain-enhancer of activated B cells (NFkB), nuclear factor erythroid 2-related factor 2 (NRF2) and extracellular signal-regulated kinase (ERK) 1/2. The release of pro-inflammatory cytokines may act as a bridge between them [5]. By doing so, mutp53 gains hyperstability, which is a prerequisite of its GOF, and contributes to chronic inflammation, a process that sustains all steps of oncogenesis [6]. The activation of unfolded protein response (UPR), which also regulates cytokine release and the activation of these pro-inflammatory and oncogenic pathways [7], may also be triggered by mutp53. Reports have shown that mutp53 can trigger the activation of the UPR sensor named activating transcription factor 6 (ATF6) [8]. This mechanism could further contribute to inflammation and the consequent activation of pathways that promote mutp53 stabilization in a vicious circle. However, mutp53 stability is also increased by the tendency of these proteins to form aggregates [9] that cannot be degraded through proteasome, a route through which wtp53 is normally degraded [10]. The inhibition of autophagy by the activation of pathways such as mTOR [11] and STAT3 by mutp53 [12,13] also contributes to increasing its expression level by reducing the possibility of being degraded through the other main catabolic route represented by lysosomes. We and other authors have recently shown that, other than macroautophagy, mutp53 can be degraded via chaperone-mediated autophagy (CMA) during endoplasmic reticulum (ER) stress [14,15]. Thus, it will be interesting to evaluate if mutp53 can manipulate this process to avoid its degradation. Other important actors that sustain mutp53 stability are represented by some members of the heat shock proteins (HSPs) family, particularly HSP90, which is known to establish a cross-talk with mutp53, either directly [16] or indirectly, through the interplay with histone deacetylase (HDAC) 6 [17]. HSPs are often upregulated in cancer cells compared to normal cells, which helps them adapt to stressful conditions in which the former are forced to live [18,19]. However, other than mutp53, HSPs stabilize a variety of oncogenic molecules and proteins involved in single or double strand brake DNA repair pathways, which is particularly important for cancer cells, as DNA damage frequently occurs in them due to the high replication rate [20,21]. 

Notably, both wtp53 and mutp53 may be decorated by several post-translational modifications (PTMs), which may have a strong impact on the expression and functions of these proteins. As most PTMs do not occur in the p53 DNA binding domain (DBD), in which mutations mainly take place, they are retained in both wt- and mutp53, with no discrimination between the two. Through mass-spectrometry analysis, 222 PTMs have been identified, occurring on 99 residues of p53 [22]. Intriguingly, the consequence of these PTMs in the regulation of wt- or mutp53 proteins may be different and, in some cases, even opposite. The most common PTMs of p53 are represented by ubiquitination, phosphorylation, acetylation, and methylation. p53 was one of the first non-histone proteins discovered to be regulated by acetylation and methylation. This discovery opened a new scenario in which enzymes responsible for these changes have emerged that also target non-histone proteins. Existing literature that reports on how PTMs may affect mutp53 compared with wtp53 will be discussed in the following paragraphs of this review. As a general concept, it is believed that phosphorylation and acetylation induce wtp53 activation, ubiquitination inhibits it, and methylation may lead to both effects, depending on the different methylation marks [23]. However, there are exceptions to this rule, and it should also be considered that PTMs influence each other, as a cross-talk occurs between them. This interplay adds another layer of complexity to the regulation of wt- and mutp53 by PTMs. Regarding mutp53, the consequences of PTMs and the outcome of the cross-talk between them are further away from being elucidated compared to wtp53. Another important aspect that will be addressed in this review is how p53 can regulate the expression and function of enzymes mediating epigenetic changes. This review also examines how these epigenetic changes could influence acetylation and methylation of histones and non-histone proteins, including p53 itself, as well as methylation of DNA and microRNAs (miRNAs) expression.

## 2. Mutp53, Ubiquitination and HSPs

It has been demonstrated that wtp53 can be degraded by mouse double minute 2 (MDM2) ubiquitin ligase and that p53 mutants, although still interacting with MDM2, can evade MDM2-mediated ubiquitination and degradation, at least in the context of tumor cells [24]. An important mechanism that prevents mutp53 ubiquitin-mediated degradation in these cells is the interplay between p53 mutant proteins and the chaperone HSP90. The latter can also cooperate with other HSPs, such as HSP70, in sustaining the stabilization of mutp53 and inhibiting its degradation, either that mediated by MDM2 and C-terminus of Hsc70-interacting protein (CHIP) E3 ubiquitin ligase [24]. However, the capacity of HSP90 to stabilize mutp53 can be strongly reduced by hyper-acetylation, as it may occur following treatment by HDAC6 inhibitors. This highlights an important strategy that may efficiently induce the downregulation of mutp53 [25]. CHIP-mediated ubiquitination and degradation of mutp53 may also be prevented by an HSP40 family protein called DnaJ Heat Shock Protein Family (HSP40) Member A1 (DNAJA1). In this regard, it has been reported that the targeting of the mevalonate pathway/DNAJA1 axis may counteract the DNAJA1-mediated stabilization of mutp53 and induce its degradation [26].

**Figure 1:**
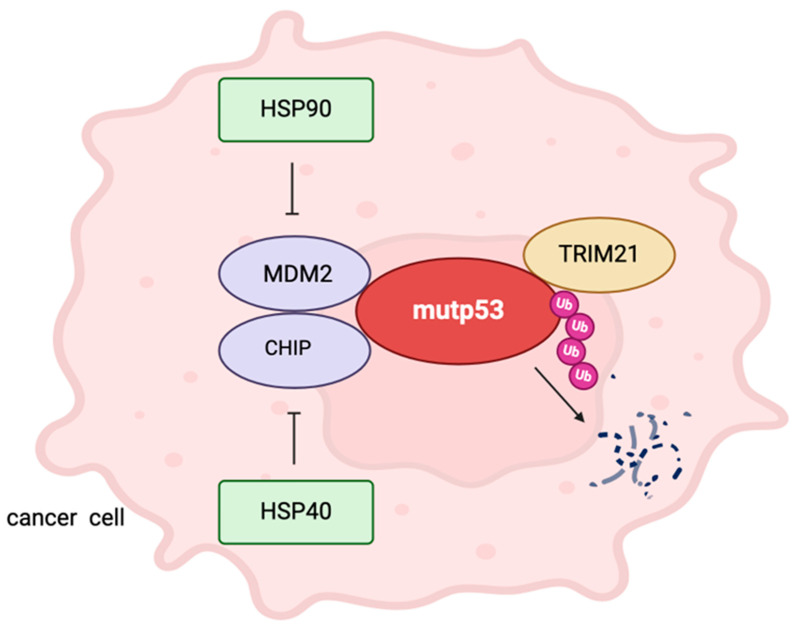
Ubiquitin ligases interacting with mutant p53 (mutp53) and heat shock proteins (HSPs) can prevent ubiquitination and proteasomal degradation of mutp53.

Among the strategies aimed at impairing mutp53 stability, we have recently discovered that the inhibition of c-Myc may promote mutp53 degradation by leading to the downregulated expression of mevalonate kinase (MVK), a kinase belonging to the mevalonate pathway [27]. However, the screening for specific mutp53-interacting proteins has unveiled that the human tripartite motif 21 (TRIM21), another E3 ubiquitin ligase, can interact with mutp53 and specifically degrade it (Figure 1). This is particularly important also because this ubiquitin ligase can spare wtp53 from degradation [28]. According to these experimental findings, TRIM21 has been found to be downregulated in most tumor cells, and its downregulation is associated with more aggressive forms of disease [28]. This suggests that strategies potentiating the expression/function of TRIM21 or searching for other ubiquitin ligases able to degrade mutp53 or investigating how to manipulate proteins stabilizing it may help to obtain this important goal and open new avenues in the treatment of cancers harboring mutp53. 

## 3. Mutp53 and Phosphorylation

**Figure 2:**
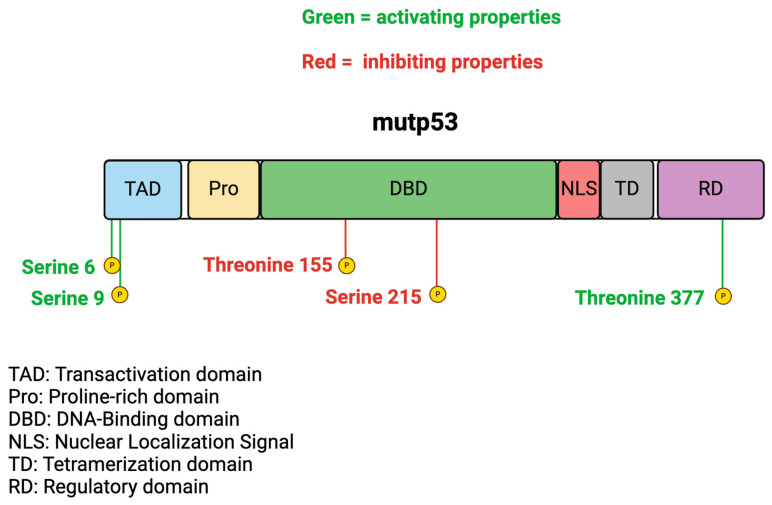
Example of phosphorylation that may sustain (serine 6, serine 9, and threonine 377) or inhibit (threonine 155 and serine 215) mutp53 activity.

Phosphorylation can strongly influence wtp53 activity, as this protein is phosphorylatable at multiple serine and threonine residues. Several kinases, including homeodomain interacting protein kinase 2 (HipK2), p38 MAP kinase, checkpoint kinase 1 (CHK1,) and c-Jun N-terminal kinase (JNK) [29], are able to phosphorylate wtp53. This process finely regulates the transcription of the different targets from the pro-survival ones, such as in the case of serine 15 phosphorylation, which mainly promotes p21 transcription and induces cell cycle arrest, to serine 46 phosphorylation, which triggers apoptosis [30]. Other than wtp53, phosphorylation may affect mutp53, and PTM also influences its function depending on the residues that undergo phosphorylation and the cellular context in which the phosphorylation occurs. It has been reported, for example, that mutp53 phosphorylation on serine 6, serine 9, and threonine 377 positively influences its GOF. Conversely, phosphorylation on threonine 155 and serine 215 may negatively affect mutp53 expression level and reduce its oncogenic activities [31,32] (Figure 2). These data suggest that strategies able to activate or inhibit kinases that affect mutp53 phosphorylation at the different sites may have a strong impact on the activity of these oncogenic proteins.

## 4. Mutp53 and Acetylation

Acetylation of histone and non-histone proteins is regulated by enzymes that add acetyl groups (writers), such as p300, p300/CBP-associated factor (pCAF), and monocytic leukemia zinc finger protein (MOZ), called acetyltransferases and those that remove acetyl groups (erasers) such as HDACs (class I, IIa, IIb, and III also called sirtuins (SIRTs) [33]. Previous studies have shown that the targeting of HDAC6, a histone deacetylase belonging to class IIb HDACs, can decrease mutp53 expression level by inducing MDM2 and inhibiting HSP90-mutant p53 complex formation [34]. The authors of this study have also shown that HDAC6 inhibition resulted in the acetylation of mutp53, but the consequences of this PTM on mutp53 stability have not been completely elucidated in this study. Later on, the silencing of transformation/transcription domain-associated protein (TRRAP), a constituent of several histone acetyltransferase (HAT) complexes, has been shown to promote mutp53 degradation via the MDM2-proteasome axis in lymphoma cells [35]. Interestingly, TRRAP is an essential cofactor for oncogenic transcription factors such as c-Myc and E1A/E2F; thus, manipulating it may have multiple consequences in cancer cells [36]. Acetyltransferases such as cAMP-response-element-binding protein (CBP)/p300 can also influence both c-Myc and p53 acetylation [37,38].

In a recent study, the role of acetylation in the regulation of mutp53 and wtp53 has been deeply investigated. The authors have evidenced that the acetylation at multiple lysines, including K373, K381, and K382, halted the missense mutated p53 (R175) aggregation, driving this protein to ubiquitination and degradation while enhancing the stability of wtp53. This has been demonstrated by several strategies, including the use of the acetylation mimic mutant in the C-terminal domain (CTD), in which these lysine residues (K) were converted to glutamine (Q) [39].

Interestingly, in pancreatic ductal adenocarcinoma cells, it has been shown that HDAC1 and 2 can regulate mutp53 expression independently of their deacetylating activity by binding to the promoter of the mutp53 gene and increasing mutp53 mRNA [40]. The findings reported above suggest that manipulating acetyltransferases and deacetylases involved in mutp53 regulation may help to reduce its expression level and, in some cases, may also reactivate the wtp53 function. The reduction of mutp53 by increasing acetylation may also be achieved because this PTM can influence the function of HSPs, such as HSP90, as mentioned above. Moreover, in cancer cells that do not harbor mutations in p53 gene, acetylation may also represent an efficacious anti-cancer strategy because acetylation has been shown to be required for the activation of wtp53 and in response to DNA damage [41].

However, the results obtained in a different study show that the acetyltransferase p300 engages a cross-talk with both wt- and mutp53 (either the conformational mutants R175H, V143A, and R249S, and the DNA contact mutants R273H and R248W) that induces the autoacetylation of p300, resulting in the activation of wtp53 transcriptional targets and sustaining mutp53 GOF as well [42].

Interestingly, other than wtp53, p300 has been reported to contribute to the stabilization of its negative regulator MDM2, thereby enhancing the p53/MDM2 negative regulatory loop [43]. Other authors have shown that CBP/p300 promotes wt- and mutp53 acetylation, activating wtp53 and restoring the wtp53 function in mutp53-carrying prostate cancer cells [44]. Similarly, the acetyltransferase PCAF can mediate the acetylation of mutp53 and reactivate the wtp53 functions [45].

**Figure 3:**
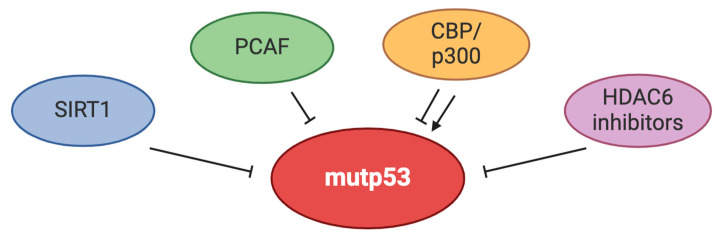
Enzymes regulating mutp53 acetylation and the outcome of this PTM on its function.

Notably, p300 can promote the formation of p53 aggregates independently of the acetyltransferase activity [46], which renders even more complex the understanding of the mechanisms through which this acetyltransferase can regulate wt- and mutp53. Last but not least, among the molecules able to affect p53 acetylation are class III histone deacetylases, namely sirtuins (SIRTs). In particular, SIRT1 has been reported to deacetylate wtp53 in a NAD+-dependent manner, inhibiting its transcriptional activity. This protects the cells from p53-dependent apoptosis or senescence but, on the other hand, predisposes to neoplastic transformation. Thus, the reduction of SIRT1 expression may represent a protective mechanism to maintain tissue homeostasis and prevent oncogenesis. Interestingly, SIRT1 activation occurs in aged cells in response to DNA damage. The role of SIRT1 in aging and tumorigenesis, acting as a tumor suppressor or tumor promoter, seems to be influenced by the intracellular localization and the cell types [47]. Regarding mutp53, it has been reported that SIRT1 activation by the small molecule called YK-3-237 deacetylates mutp53, leading to its depletion and upregulating the expression of wtp53-targets Puma and Noxa, in triple-negative breast cancer (TNBC) cells [48]. Altogether, these findings highlight the potential of manipulating acetylation as a strategy to inhibit mutp53 but also demonstrate the complexity of the regulation of wt- and mutp53 activities by this PTM (Figure 3).

## 5. Methylation and wt- and mutp53

Protein methylation is mediated by enzymes that add from one to three methyl groups on particular substrates, the lysine ε-amino group of histone or non-histone proteins. By accepting different numbers of methyl groups, the proteins may undergo mono-, di-, or trimethylation (me1, me2, or me3), resulting in a different outcome on gene transcription [49]. On the other hand, enzymes that remove methyl groups oppositely influence this PTM. Several transcription factors can undergo methylation/demethylation at different lysine sites and to different degrees. Among them, wtp53 may be decorated by lysine methylation at multiple sites, which regulates its activity. For example, mono-methylation of p53 at lysine 372 (p53K372me1), which is mediated by the SET domain-containing lysine methyltransferase 7 (also called Set9 or SET7/9) and takes place in the nucleus, positively affects p53 stability as well as the expression of p53 target genes [50]. Interestingly, SET7/9 stabilizes chromatin-bound wtp53 by positively influencing its acetylation [51]. Furthermore, SET7/9 has been shown to abrogate the de-acetylating activity of SIRT1 on wtp53 [52]. Differently from SET7/9, mono-methylation of wtp53 at lysine 370 (p53K370me1), mediated by the lysine methyltransferase SET and MYND domain-containing protein 2 (Smyd2), results in the repression of its transactivating activity [53].

Lysine mono-methylation of p53 at lysine 382 (p53382me1), induced by the methyltransferase SET8, also reduces p53-mediated transcription of the highly responsive target genes [54].

**Figure 4:**
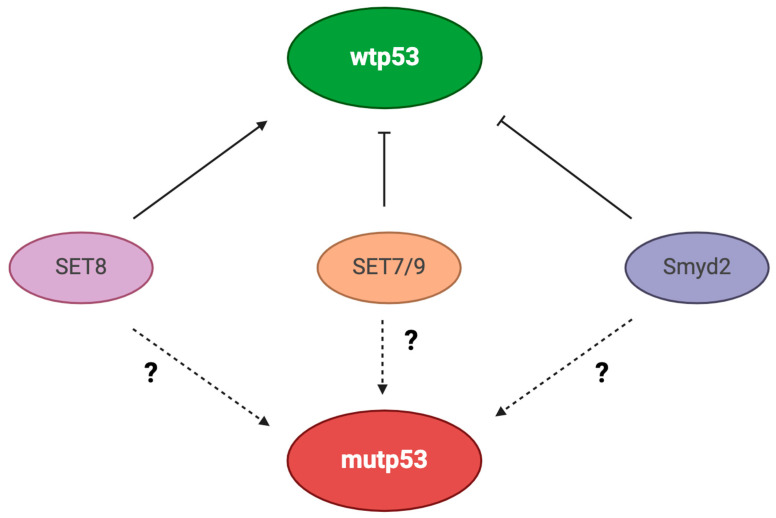
Enzymes affecting p53 methylation, positively (SET8) or negatively (SET7/9 and Smyd2) regulate its function.

As stated above, other than methyltransferases, several demethylases can control p53 methylation. In particular, it has been reported that lysine (K)-specific demethylase 1 (KDM) 1 (also called LSD1) interacts with wtp53 and demethylates it at K370, leading to either mono or dimethylation of wtp53 and repressing its function [55]. KDM4C, the histone H3K9 demethylase, can also demethylate wtp53, but it activates rather than inhibits its pro-apoptotic functions. Interestingly, KDM4C may also act on c-Myc, downregulating its expression [56]. KDM1 is a c-Myc transcriptional target [57], which implies that c-Myc can indirectly methylate wtp53 through KDM1, reducing its activity. These studies suggest that changes in methylation can result in activation or inhibition of wtp53, strongly influencing the outcome of anti-cancer therapies and tumor prevention (Figure 4).

If several studies, including those reported above, have explored the effects of lysine methylation on wtp53 functions, the impact of methylation at these lysine residues (e.g., K372, K382, and K370) in the regulation of mutp53 stability and GOF, remains to be clarified and may represent an interesting topic to be investigated in the search for new therapeutic approaches to fight cancer carrying mutp53. Furthermore, the same lysine other than methylation can undergo acetylation, and an interplay between them and other PTMs has been documented, which together influence transcription in basal conditions and in response to stress and DNA damage.

## 6. Mutp53 and Epigenetic Changes

Epigenetic changes are heritable changes in the genome that occur independently of gene mutations. They include DNA methylation and histone modifications, such as acetylation and methylation, and non-coding RNA regulations. Notably, epigenetic alterations, together with genetic mutations and environmental factors, are the main factors contributing to cancer onset [58]. These modifications have been shown to be interconnected. Interestingly, several studies have evidenced that mutp53 may influence epigenetics [59] because, for example, it may alter the expression of enzymes mediating acetylation and methylation. Regarding acetylation, as mentioned in the above paragraph, both wtp53 and mutp53 can activate the acetyltransferase p300, resulting in an increase in p300 acetylation [42]. However, hyper-acetylation of p300 may contribute to p53 activation and regulates other proteins involved in oncogenesis, such as c-Myc [37]. Considering that c-Myc may, in turn, influence mutp53 stability [27] and establish cross-talks with wtp53 [60], the acetylation of c-Myc can indirectly influence both wt- and mutp53.

Regarding methylation, a recent paper has reported that the polycomb-group histone methyltransferase enhancer of zeste homolog 2 (EZH2), which mainly inhibits gene transcription by tri-methylating histone H3 on lysine 27 (H3K27me3), can also influence p53, independently on H3K27me3 function, by binding to p53 mRNA. Activated wtp53 may repress the promoter of EZH2, an effect dependent on p21 target transcription [61], while EZH2 depletion enhances wtp53 stabilization through the de-repression of cyclin-dependent kinase inhibitor 2A (CDKN2A) [62]. These data highlight a complex functional interaction between EZH2 and wtp53, which deserves further investigation. Regarding mutp53, it has been shown that its expression can be increased by EZH2, and this effect seems to occur independently of the methyltransferase activity of this enzyme [63]. Interestingly, mutp53 has been reported to increase the expression of EZH2, which further sustains oncogenesis [64]. This methyltransferase plays a key role in the different steps of carcinogenesis, from cancer onset to cancer progression. Therefore, it emerges that the positive feedback loop that mutp53 establishes with it is an important pro-tumorigenic effect. It can be expected that the upregulation of a methyltransferase such as EZH2 by mutp53 may result in methylation changes of histones and in aberrant regulation of gene transcription, impacting, for example, the expression of tumor suppressors, with important consequences on cancer cell biology. Notably, EZH2 has been reported to activate STAT3, and this could be a mechanism through which mutp53 supports the activation of this oncogenic pathway [13]. EZH2 results mutated in mutp53 carrying cancer cells or upregulated because of the absence of MDM2, as it, together with murine double minute X (MDMX), targets EZH2 for ubiquitination and degradation [65]. In addition to EZH2, other methyltransferases, such as lysine methyltransferase (KMT) 2A (MLL1) and KMT2D (MLL2), have been reported to be upregulated by mutp53, in some cases concomitantly to the acetyltransferase MOZ [66]. Moreover, p53 mutants can interact with supervillin (SVIL) and recruit the H3K4me3 methyltransferase MLL1, activating the expression of YTHDF2 N6-methyladenosine (m6A) reader, hampering the expression of m6A-marked tumor-suppressing transcripts and supporting gliomagenesis [67]. Furthermore, mutp53 can cooperate with the histone mono-methyltransferase MLL4, modulating aberrant enhancer activity and promoting tumor gene expression [68]. As discussed above, previous studies have shown that mutp53 can induce the transcription of enzymes able to mediate acetylation, including MOZ and p300, which may alter the acetylation of histones and non-histone proteins, besides p53 itself. Based on these findings, it emerges that wt- and mutp53 have a high potential to alter the methylation and acetylation landscape, which may be another mechanism driving oncogenesis.

Last but not least, DNA methylation may be affected by mutp53, as it has been reported that p53 mutant proteins lose the suppressive activity toward DNA (cytosine-5)-methyltransferase 1 (DNMT1) mediated by wtp53, thus enhancing DNA methylation and leading for example to the downregulation of oncosuppressors such as p16 (ink4A) [69]. The expression of other molecules involved in anti-cancer surveillance could be affected in the same manner by DNA hypermethylation. However, it is emerging that depending on the localization in the promoter regions or in the gene body, this epigenetic modification may lead to a different effect, mainly repressing the transcription in the first case and activating it in the latter case [70].

## 7. Mutp53 and miRNAs

Depending on the specific mutations, p53 may unbalance the expression of several miRNAs, resulting in profound changes in cell proliferation, differentiation, and apoptosis. miRNAs are non-coding RNAs of about 20–25 nucleotides in length, whose expression is highly dysregulated in cancer cells, with a high impact on the post-transcriptional regulation of gene expression [71]. In the case of colon cancer cells, among the 376 mature miRNAs evaluated, mutp53R273H has been found to downregulate 33 miRNAs and upregulate four of them [72]. This reflects the general decrease of mature miRNAs frequently observed in tumors in correlation with defects of their post-transcriptional maturation rather than perturbation of transcription. However, the miRNAs more often downregulated by mutp53, not only by hot-spot mutants p53R273H and p53R175H, is miR-517a, which leads to cell cycle arrest in G2/M and triggers an apoptotic cell death in colon cancer [72]. Another miRNA reduced by several p53 mutants is miR-27a, which induces an increase in epidermal growth factor receptor (EGFR) expression and promotes cancer proliferation [73]. Other miRNAs downregulated by mutp53 include miR-130b, miR-223, miR-218, and miR-519a, which have been shown to contribute to cancer survival and progression. However, several of these miRNAs have been reported to play a dual role in cancer, promoting or reducing carcinogenesis, depending on the specific cellular context [74]. The expression of several miRNAs regulated by mutp53 can be influenced in an opposite way by wtp53, for example, in the case of miR-26a-1, whose maturation is increased by wtp53 and reduced by mutp53. As EZH2 can be considered a target of this miRNA, this results in an opposite regulation of histone methylation and changes in the expression of several tumor suppressor genes or oncogenes by wt- and mutp53 [64].

Regarding the miRNAs upregulated by mutp53, miR-128-2 has been shown to be upregulated by p53R175H mutant in lung cancer and leading to an increase of chemoresistance [75], and miR-155, upregulated by p53R248Q or p53R282W mutants in bladder and gastric cancers, in correlation with poor prognosis of the disease. Among the other oncogenic properties of miRNA 155, it has been reported to promote epithelial-mesenchymal transition (EMT) by affecting the transforming growth factor (TGF)-β pathway [76]. miR-21 can also be upregulated by mutp53, enhancing the secretion of exosomes by cancer cells, with important implications in the communication between cancer cells and cells of the tumor environment, such as immune cells, fibroblasts, and endothelial cells.

## 8. Conclusions

The studies performed on wtp53 in the last 40 years have elucidated multiple pathways and mechanisms by which this protein may be modified at more than 60 sites. Only more recently have studies focused on PTMs that may decorate the mutant forms of p53. This is important because PTMs might offer a promising opportunity to regulate the key functions that wt- and mutp53 play in carcinogenesis. However, it appears that the field is complex because PTMs are numerous, and the effects that they induce may vary depending on cell context. To render this even more complicated this landscape, it is emerging that PTMs are interconnected. For example, acetylation may be influenced by phosphorylation, methylation, and ubiquitination, and acetylation can, in turn, influence them, resulting in a fine modulation of p53 activity. Last but not least, we highlighted several studies reporting that wt- and mutp53 may have an important impact on epigenetic regulation. Thus, a better understanding of this aspect may have important implications on the expression and function of proteins involved in cancer.

## Data Availability

Not applicable.
